# Interplay between Phenotypic Resistance to Relevant Antibiotics in Gram-Negative Urinary Pathogens: A Data-Driven Analysis of 10 Years’ Worth of Antibiogram Data

**DOI:** 10.3390/life11101059

**Published:** 2021-10-08

**Authors:** Márió Gajdács, Zoltán Bátori, Katalin Burián

**Affiliations:** 1 Department of Oral Biology and Experimental Dental Research, Faculty of Dentistry, University of Szeged, Tisza Lajos krt. 63., 6720 Szeged, Hungary; 2 Department of Ecology, Faculty of Sciences, University of Szeged, Közép fasor 52., 6726 Szeged, Hungary; zbatory@gmail.com; 3 Department of Medical Microbiology, Albert Szent-Györgyi Health Center, Faculty of Medicine, University of Szeged, Semmelweis utca 6., 6725 Szeged, Hungary; burian.katalin@med.u-szeged.hu

**Keywords:** urinary tract infection, Gram-negative, antimicrobial drug resistance, MDR, surveillance, big data, principal component analysis, correlation matrix

## Abstract

The global emergence of antimicrobial resistance (AMR) has become a critical issue for clinicians, as it puts the decades of developments in the medical field in jeopardy, by severely limiting the useful therapeutic arsenal of drugs, both in nosocomial and community-acquired infections. In the present study, a secondary analysis of taxonomic and resistance data was performed, corresponding to urinary tract infections (UTIs) caused by Gram-negative bacteria, detected between 1 January 2008 to 31 December 2017 at the Albert Szent-Györgyi Health Center, University of Szeged. The following were identifiable from the data collected: year of isolation; outpatient (OP)/inpatient (IP) origin of the isolate; taxonomy; and susceptibility/resistance to selected indicator antibiotics. Principal component analysis (PCA) and a correlation matrix were used to determine the association between the presences of resistance against indicator antibiotics in each taxonomic group. Overall, data from *n* = 16,240 outpatient and *n* = 13,964 inpatient Gram-negative UTI isolates were included in the data analyses. In *E. coli*, strong positive correlations were seen between resistance to ciprofloxacin (CIP) and gentamicin (GEN) resistance (OP: r = 0.6342, *p* = 0.049; IP: r = 0.9602, *p* < 0.001), whereas strong negative correlations were shown for fosfomycin (FOS) and nitrofurantoin (NIT) resistance (OP: r = −0.7183, *p* = 0.019; IP: r = −0.7437; *p* = 0.014). For *Klebsiella* spp. isolates, CIP resistance showed strong positive correlation with resistance to third-generation cephalosporins (3GC) and GEN (r = 0.7976, *p* = 0.006 and r = 0.7428, *p* = 0.014, respectively) in OP isolates, and with resistance to trimethoprim-sulfamethoxazole (SXT) and FOS (r = 0.8144, *p* = 0.004 and r = 0.7758, *p* < 0.001, respectively) in IP isolates. For members of the *Citrobacter-Enterobacter-Serratia* group, the resistance among indicator antibiotics showed a strong positive correlation, with the exception of FOS resistance. In the *Proteus-Providencia-Morganella* group, the strongest association was noted between CIP and SXT resistance (OP: r = 0.9251, *p* < 0.001; IP: r = 0.8007; *p* = 0.005). In the case of OP *Acinetobacter* spp., CIP showed strong and significant positive correlations with most indicator antibiotics, whereas for IP isolates, strong negative correlations arose among imipenem (IMI) resistance and resistance to other drugs. For *Pseudomonas* spp., strong and positive correlations were noted among resistance to β-lactam antibiotics and aminoglycosides, with the exception of ceftazidime (CEFT), showing strong, but negative correlations. Though molecular tests and sequencing-based platforms are now considered as the gold-standard for AMR surveillance, standardized collection of phenotypic resistance data and the introduction of Big Data analytic methods may be a viable alternative for molecular surveillance, especially in low-resource settings.

## 1. Introduction

The introduction of antibiotics has ushered in a new age of modern medicine, allowing for the successful treatment of previously life-threatening infections, and for the development of many complex medical fields and specialties [1,2]. The global emergence of multidrug resistant (MDR) bacteria has become a critical issue for clinicians, as they put the decades of developments in the medical field in jeopardy, by severely limiting the useful therapeutic arsenal of drugs, both in nosocomial and community-acquired infections [3,4]. The clinical problem of MDR is multifaceted, including the easy access to, and often inappropriate use of, existing antibiotics (i.e., prescriptions in inappropriate indications or for inappropriate time periods, self-medication without prescription), and the lack of development and market authorization of novel antimicrobials [5,6]. Warnings have been put out for national governments by both the US and European Centers for Disease Control and Prevention (CDC, ECDC) and the World Health Organization (WHO), calling for global and intersectoral action to address these concerns [7,8,9]. In fact, the O’Neill report has estimated that by 2050, infectious diseases may lead to the death of 10 million individuals, once again becoming the second leading cause of mortality [10].

Bacteria may become resistant to antibiotics through a variety of mechanisms, and some of these mechanisms are intrinsic, i.e., they are characteristic for the genus/species in question, and resistance is passed on vertically to the daughter cells [11]. Far more commonly, resistance determinants may be found on mobile genetic elements (MGEs), including plasmids, transposons, and integrons [12]. From a public health perspective, these MGEs are especially concerning, as they may carry resistance genes for multiple antibiotics, allowing for the rapid dissemination and outbreak-formation in taxonomically-diverse bacteria [13]. Resistance-determinants encoded on plasmids have been critical in the development of the MDR phenotype in many relevant pathogens [14]. Additionally, adaptive mechanisms of resistance—including biofilm-formation, metabolic switching, and small-colony variant (SCV) formation—must also be mentioned, most often leading to phenotypic resistance in vivo [15,16]. Though methicillin-resistant *Staphylococcus aureus* (MRSA) may be considered as the first MDR “superbug”, after the 2000s, a pronounced shift has been observed towards the relevance of MDR Gram-negative bacteria [15]. This is due to the emergence isolates presenting with extended-spectrum β-lactamases (ESBLs), carbapenem-resistance (either due to carbapenemases or membrane impermeability) and even colistin-resistance, and the lack of appropriate antimicrobials to treat these infections [17,18].

Urinary tract infections (UTIs) are the second most common infections in developed countries, leading to ~7–8 million visits to primary care physicians and over 1 million visits to the Emergency Department in the US alone [19,20]. UTIs also account for substantial economic costs (~3–5 billion US dollars) worldwide, associated with treatment, hospital costs, and productivity losses [21]. Hospitalization rates due to UTIs have also shown an increasing trend worldwide [22]. Although females of a reproductive age are the most commonly burdened with uncomplicated UTIs, the disease may affect anyone, irrespective of age, gender, or socio-economic status [23,24]. The most common causative agent in in both community- and hospital-associated UTIs is *Escherichia coli*, whereas Gram-negative bacteria are responsible for 80–90% of all UTIs overall [25]. In older patients, or patients with immunosuppression or other underlying conditions, some species, such as *Proteus* spp. or non-fermenters (*Pseudomonas* spp., *Acinetobacter* spp.), are more commonly found as pathogens [26].

Resistance rates in various geographical regions may show pronounced variations, which should be monitored over time [27]. Continuous surveillance of resistance rates is of great importance, and this data may also be used to aid the introduction and evaluation of antimicrobial stewardship interventions in healthcare institutions [28]. With the advent of molecular methods, next-generation sequencing (NGS), and in-depth bioinformatics analyses in public health microbiology, it has been encouraged that the occurrence and co-occurrence of resistance in clinical isolates be determined on a genetic level, with the aim of identifying the most commonly occurring gene(s) responsible for the resistant phenotype, and to ascertain the attributes of successful MDR clones [29]. This data also sheds light on co-occurring resistance mechanisms in Gram-negative bacteria, which may be the basis of driving therapeutic choices in outpatient empirical treatment and the ambulatory use of antibiotics, to avoid the selection of highly-resistant variants [30]. Nevertheless, these methods are costly, time-consuming, not compatible with a large number of clinical isolates, and often only available in national reference laboratories. Therefore, in many clinical microbiology laboratories, reporting of resistance rates (for epidemiological purposes) from clinical isolates heavily relies on phenotypic antimicrobial susceptibility results and tests based on biochemical reactions (e.g., for carbapenemase-detection) [31]. Still, systematically-collected phenotypic resistance data over a long time period may also provide important insights on the association of resistance with clinically important antibiotic groups [32].

In our previous report, we sought to describe the epidemiological aspects and resistance rates of Gram-negative pathogens—including members of the Enterobacterales order and non-fermenters—implicated in UTIs from inpatients and outpatients in a large tertiary-care hospital in Southern Hungary. In the article, the levels of UDR (usual drug resistance), MDR, XDR (extensive drug resistance), and DTR (difficult-to-treat resistance) were calculated, in addition to the introduction of a composite multiple antibiotic resistance (pMAR) score and the modified versions (mDTR, mcDTR) of these resistance categories [33]. As there is limited data on the interplay between resistances to different antibiotic groups contributing to the multiple drug resistant phenotype, the aim of our study was to provide data in this regard from our already existing dataset, corresponding to UTIs in the Southern region of Hungary.

## 2. Materials and Methods

### 2.1. Study Design, Setting

In the present study, a secondary analysis of taxonomic and resistance data was performed, which was initially collected for epidemiological purposes [33]. The data corresponds to UTIs detected between the time period of 1 January 2008 to 31 December 2017 in outpatient clinics and inpatient departments at the Albert Szent-Györgyi Health Center, University of Szeged, a primary- and tertiary-care teaching hospital in the Southern Region of Hungary (serving ~600,000 individuals in the region [34]). Data collection was performed manually from the database of the Department of Microbiology’s (previously: Institute of Clinical Microbiology) laboratory information system (LIS), corresponding to urine samples positive for Gram-negative pathogens, based on the criteria described previously [33].

### 2.2. Bacterial Identification, Antimicrobial Susceptibility Testing

The processing of urine samples arriving at the Department of Medical Microbiology during the study period was carried out based on the relevant guidelines published by the Hungarian Ministry of Health [35]. Identification of isolates was carried out via classical biochemical tests, the VITEK 2 ID/AST (bioMérieux, Marcy-l’Étoile, France), and after 2012, matrix-assisted laser desorption/ionization time-of-flight mass spectrometry (MALDI-TOF MS) using a MicroFlex MALDI Biotyper (Bruker Daltonics, Bremen, Germany) [36]. Antimicrobial susceptibility testing for Gram-negative bacteria and the interpretation of the results was performed based on the recommendations of the European Committee on Antimicrobial Susceptibility Testing (EUCAST) at the time of isolation, taking into account the intrinsic resistance mechanisms of isolated bacteria [37]. Resistance to third-generation cephalosporins (3GCs) in Enterobacterales species was inferred from resistance against ceftriaxone and/or ceftazidime [38]. Intermediate results (I) for antibiotics other than 3GCs were grouped with and reported as resistant (R) [36].

### 2.3. Data Collection, Preparation for Data Analysis

The data were collected from the LIS and saved as Microsoft Excel 2013 (Microsoft Corporation, Redmond, WA, USA) documents for statistical analysis. For the purposes of the analyses, the following taxonomic groups were set for Gram-negative bacteria: *E. coli*; *Klebsiella* spp.; the *Citrobacter-Enterobacter-Serratia* [CES] group; the *Proteus-Providencia-Morganella* [PPM] group; *Acinetobacter* spp.; and *Pseudomonas* spp. Additionally, indicator antibiotics were selected for each taxonomic group, for which, resistance data was included in the analysis. For *Klebsiella* spp., the CES group, and the PPM group, these antibiotics were 3GCs, ciprofloxacin (CIP), gentamicin (GEN), trimethoprim-sulfamethoxazole (SXT), and fosfomycin (FOS). For *E. coli*, they were 3GCs, CIP, GEN, SXT, FOS, and nitrofurantoin (NIT). For *Acinetobacter* spp., they were CIP, GEN, amikacin (AMI), SXT, imipenem (IMI), and meropenem (MER). For *Pseudomonas* spp., they were CIP, GEN, AMI, ceftazidime (CEFT), cefepime (CEFE), IMI, and MER. Due to the low number of carbapenem-resistant Enterobacterales, and the low number of colistin non-susceptible Gram-negative isolates overall, these antibiotics were not selected for the relevant taxonomic groups [36]. The following were identifiable from the data: year of isolation; outpatient/inpatient origin of the isolate; taxonomy; and susceptibility or resistance to indicator antibiotics (5–7, depending on the taxonomic group).

### 2.4. Statistical Analyses: Principal Component Analysis (PCA), Pearson-Correlation

Descriptive statistics was performed using Microsoft Excel 2013 (Microsoft Corporation, Redmond, WA, USA). The normality of variables was tested using the Kolmogorov–Smirnov test. Principal component analysis (PCA) was applied to assess the association between pair-wise variables (resistance against indicator antibiotics) in each taxonomic group. PCA was used to reduce data dimensions and to visualize each dataset in a two-dimensional space. During this process, the data was projected on new coordinate directions, i.e., principal component 1 [PC1] and principal component 2 [PC2], where the original data has the largest variance explained, to identify the presence of individual resistance in these bacteria and their contribution to overall resistance rates [39,40]. A correlation matrix was used to determine the association between the presence of resistance against indicator antibiotics. Based on the value of the Pearson-correlation coefficients (r), the relationship between the variables was determined as follows: 0.1 < |r| < 0.3 were denoted as weak correlation; 0.3 < |r| < 0.5 as moderate correlation; 0.5 < |r| < 0.85 as strong correlation; and |r| ≥ 0.85 as very strong correlation [41]. Statistical analyses were performed using the Past 4.01 statistical software (Paleontological Museum, University of Oslo; Oslo, Norway). *p* values < 0.05 were considered statistically significant.

## 3. Results and Discussion

### 3.1. General Information

Overall, data from *n* = 16,240 outpatient and *n* = 13,964 inpatient Gram-negative UTI isolates were included in the data analysis, corresponding to our 10-year (2008–2017) study period. The distribution among different taxonomic groups was the following (n_OP_: outpatient; n_IP_: inpatient isolates): *E. coli*: n_OP_ = 12,002 and n_IP_ = 8173; *Klebsiella* spp.: n_OP_ = 1895 and n_IP_ = 2952; CES group: n_OP_ = 554 and n_IP_ = 578; PPM group: n_OP_ = 1058 and n_IP_ = 1392; *Acinetobacter* spp.: n_OP_ = 143 and n_IP_ = 133; and *Pseudomonas* spp.: n_OP_ = 588 and n_IP_ = 1096. The rates of MDR in the respective taxonomic groups were the following: *E. coli* 2.3%; *Klebsiella* spp. 1.6%; CES group 5.9%; PPM group 9.1%; *Acinetobacter* spp. 9.7%; and *Pseudomonas* spp. 8.5%. In most cases, inpatient isolates presented with significantly higher rates of resistance (*p* < 0.05) compared to outpatient isolates [33].

### 3.2. Outpatient Isolates

**Figure 1:**
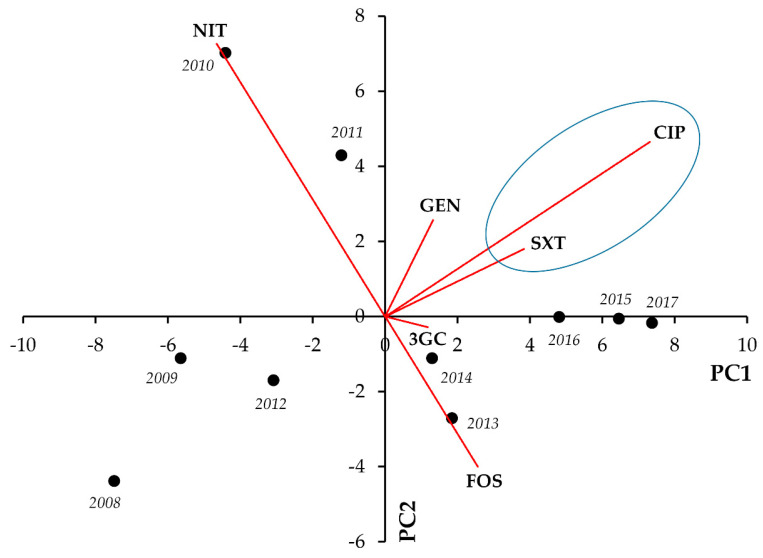
Principal component analysis (PCA) of resistance data for outpatient *E. coli* UTI isolates among six indicator antibiotics (2008–2017). PC1 and PC2 axes explained 62.05% and 25.50% of the total variance in the dataset, respectively. The blue ellipse denotes the positive correlation between the resistance to two antibiotics from a different antibiotic family with the highest Pearson-correlation coefficient.

**Figure 2:**
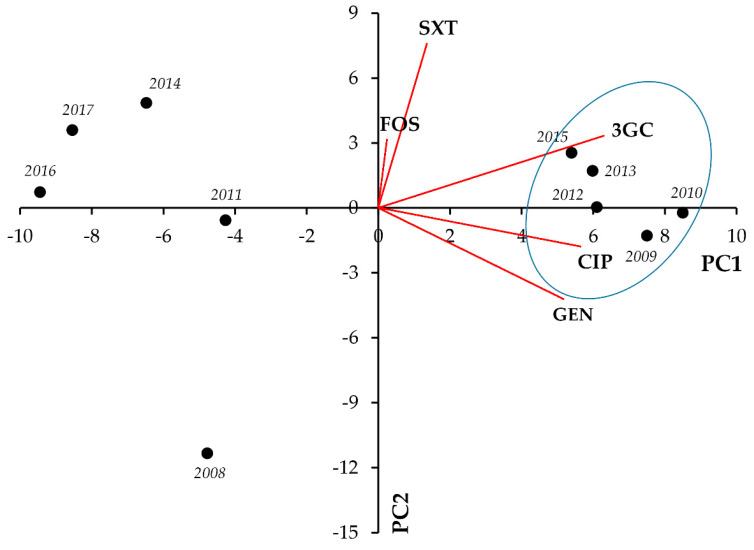
Principal component analysis (PCA) of resistance data for outpatient *Klebsiella* spp. UTI isolates among five indicator antibiotics (2008–2017). PC1 and PC2 axes explained 57.23% and 21.28% of the total variance in the dataset, respectively. The blue ellipse denotes the positive correlation between the resistance to two antibiotics from a different antibiotic family with the highest Pearson-correlation coefficient.

**Figure 3:**
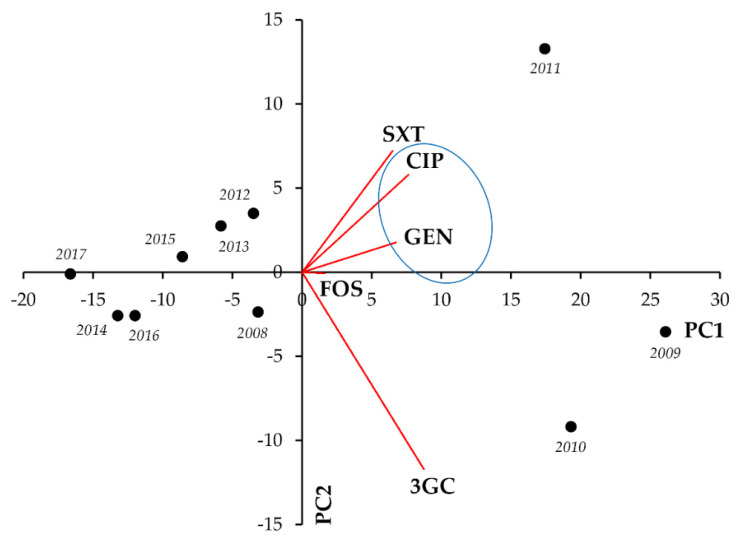
Principal component analysis (PCA) of resistance data for outpatient CES group UTI isolates among five indicator antibiotics (2008–2017). PC1 and PC2 axes explained 79.39% and 11.91% of the total variance in the dataset, respectively. The blue ellipse denotes the positive correlation between the resistance to two antibiotics from a different antibiotic family with the highest Pearson-correlation coefficient.

**Figure 4:**
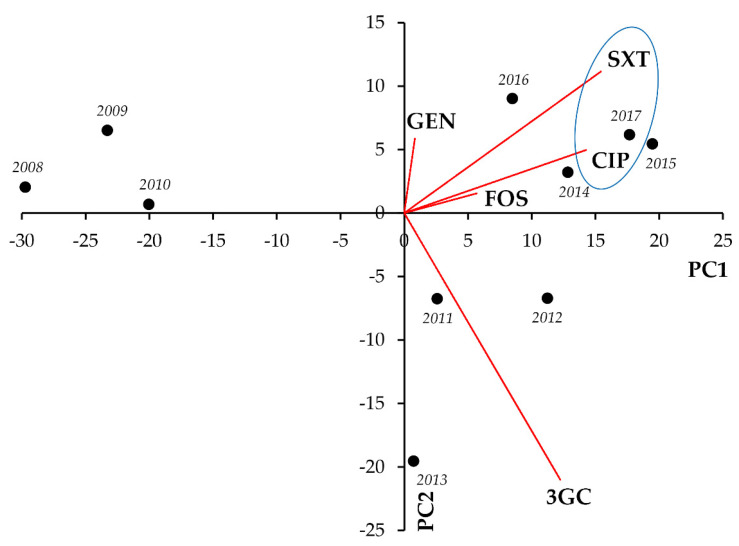
Principal component analysis (PCA) of resistance data for outpatient PPM group UTI isolates among five indicator antibiotics (2008–2017). PC1 and PC2 axes explained 77.01% and 18.09% of the total variance in the dataset, respectively. The blue ellipse denotes the positive correlation between the resistance to two antibiotics from a different antibiotic family with the highest Pearson-correlation coefficient.

**Figure 5:**
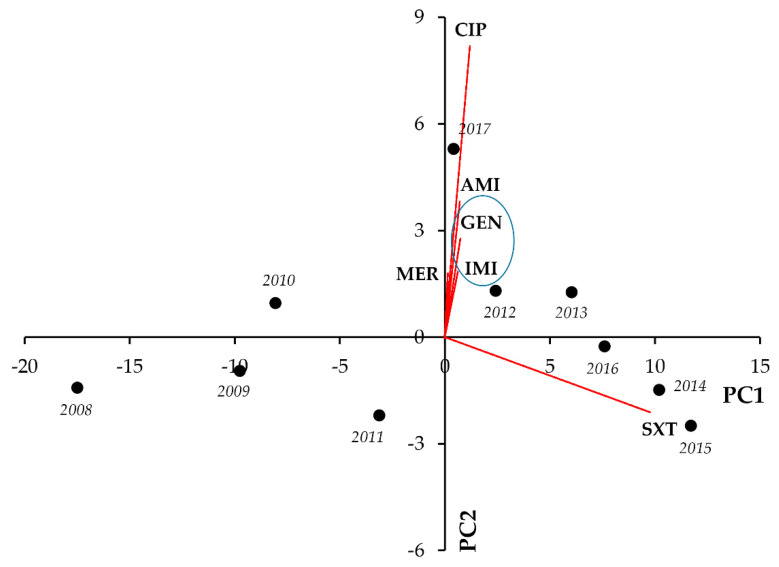
Principal component analysis (PCA) of resistance data for outpatient *Acinetobacter* spp. UTI isolates among six indicator antibiotics (2008–2017). PC1 and PC2 axes explained 92.66% and 5.50% of the total variance in the dataset, respectively. The blue ellipse denotes the positive correlation between the resistance to two antibiotics from a different antibiotic family with the highest Pearson-correlation coefficient.

**Figure 6:**
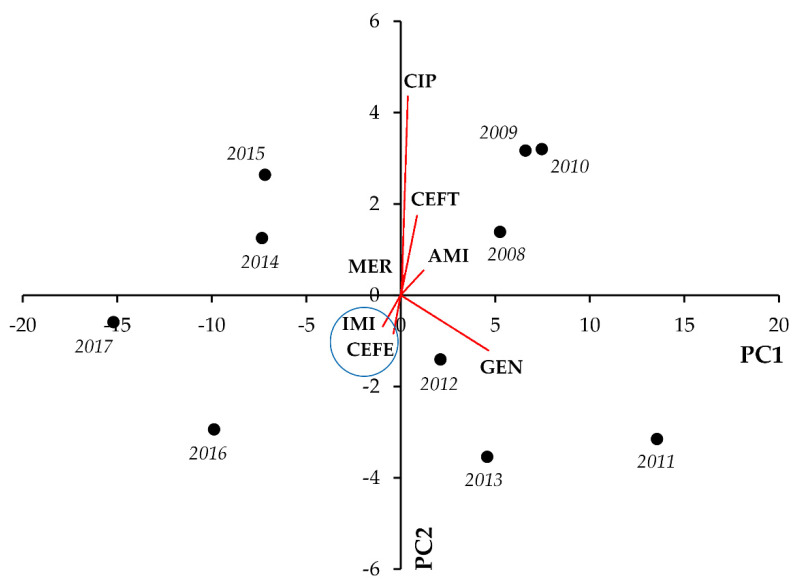
Principal component analysis (PCA) of resistance data for outpatient *Pseudomonas* spp. UTI isolates among seven indicator antibiotics (2008–2017). PC1 and PC2 axes explained 85.56% and 7.12% of the total variance in the dataset, respectively. The blue ellipse denotes the positive correlation between the resistance to two antibiotics from a different antibiotic family with the highest Pearson-correlation coefficient.

The results of the PCA analyses and the corresponding correlation matrices for the data from outpatient UTI isolates are presented in Figure 1, Figure 2, Figure 3, Figure 4, Figure 5 and Figure 6 and Table 1, Table 2, Table 3, Table 4, Table 5 and Table 6, respectively. In *E. coli*, strong positive correlations were seen between CIP-SXT (r = 0.8474; *p* = 0.002) and CIP-GEN resistance (r = 0.6342; *p* = 0.049), whereas strong negative correlation was shown for FOS and NIT resistance (r = −0.7183; *p* = 0.019) (Figure 1, Table 1).

In *Klebsiella* spp., strong positive correlation was seen between CIP-3GC (r = 0.7976; *p* = 0.006) and CIP-GEN resistance (r = 0.7428; *p* = 0.014) (Figure 2, Table 2).

For members of the *Citrobacter-Enterobacter-Serratia* group, the existence of resistance to most indicator antibiotics showed strong and significant positive correlations (the strongest being GEN-CIP [r = 0.9326; *p* < 0.001], GEN-SXT [r = 0.8152; *p* = 0.004], and GEN-3GC [r = 0.8144; *p* = 0.004], respectively), whereas such strong associations were not shown between resistance to FOS and other antimicrobials (Figure 3, Table 3).

For members of the *Proteus-Providencia-Morganella* group, strong and significant positive correlations were seen between FOS-CIP (r = 0.8656; *p* < 0.001), FOS-SXT (r = 0.8656; *p* < 0.001), and CIP-SXT (r = 0.9251; *p* < 0.001) (Figure 4, Table 4).

In the case of *Acinetobacter* spp., CIP showed strong and significant positive correlations with most indicator antibiotics (apart from SXT, which showed strong positive associations with the aminoglycosides GEN and AMI). In addition, GEN-IMI and GEN-AMI co-resistance presented with very strong and significant correlations (r = 0.8620, *p* = 0.001, and r = 0.9838, *p* < 0.001, respectively) (Figure 5, Table 5).

For *Pseudomonas* spp., most notable positive correlations were seen between CEFE and IMI (r = 0.8617; *p* = 0.001), MER (r = 0.7338; *p* = 0.016) and GEN (r = 0.6857; *p* = 0.028) resistance, and GEN-AMI resistance (r = 0.8745; *p* < 0.001). On the other hand, strong negative correlations arose among CEFT-IMI (r = −0.7879; *p* = 0.007), CEFT-CEFE (r = −0.8376; *p* = 0.002), and GEN-IMI (r = −0.6595; *p* = 0.038) (Figure 6, Table 6).

### 3.3. Inpatient Isolates

**Figure 7:**
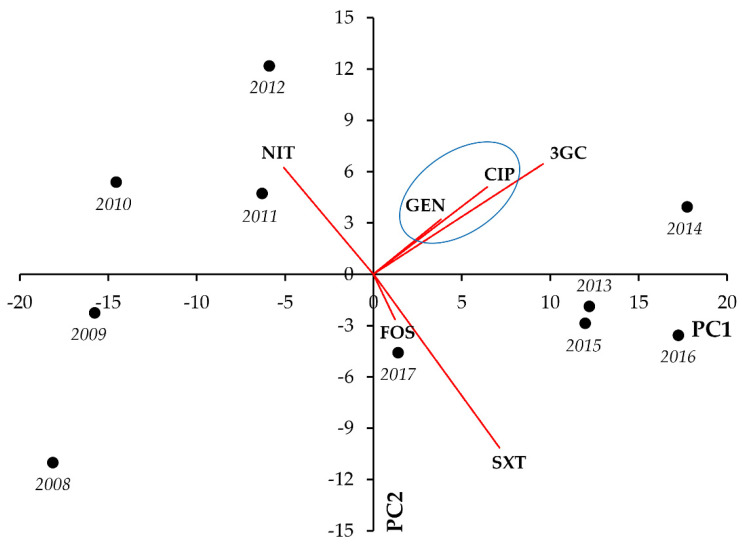
Principal component analysis (PCA) of resistance data for inpatient *E. coli* UTI isolates among six indicator antibiotics (2008–2017). PC1 and PC2 axes explained 68.39% and 14.97% of the total variance in the dataset, respectively. The blue ellipse denotes the positive correlation between the resistance to two antibiotics from a different antibiotic family with the highest Pearson-correlation coefficient.

**Figure 8:**
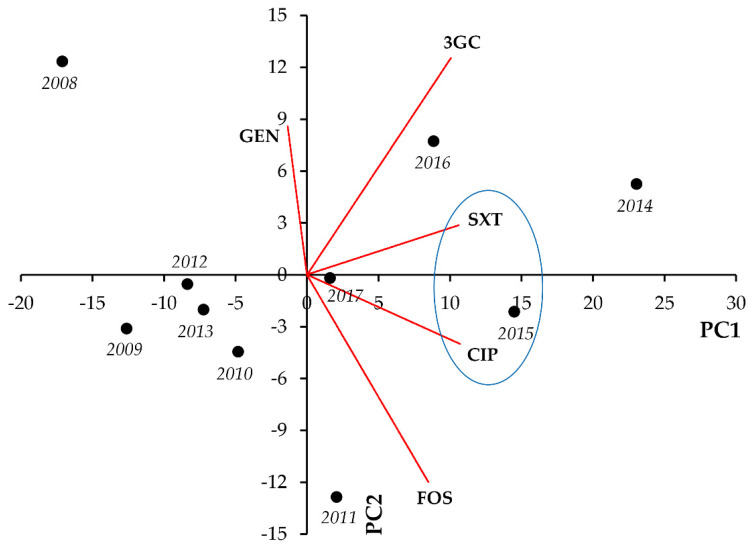
Principal component analysis (PCA) of resistance data for inpatient *Klebsiella* spp. UTI isolates among five indicator antibiotics (2008–2017). PC1 and PC2 axes explained 66.42% and 20.71% of the total variance in the dataset, respectively. The blue ellipse denotes the positive correlation between the resistance to two antibiotics from a different antibiotic family with the highest Pearson-correlation coefficient.

**Figure 9:**
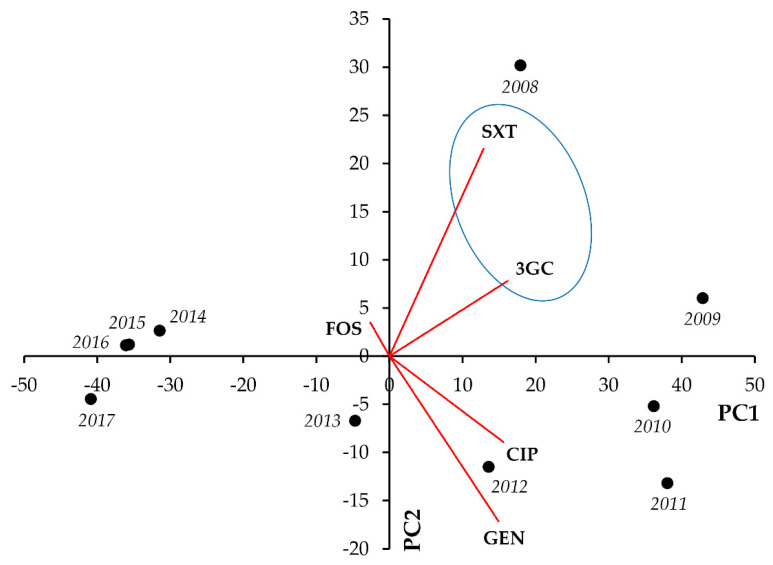
Principal component analysis (PCA) of resistance data for inpatient CES group UTI isolates among five indicator antibiotics (2008–2017). PC1 and PC2 axes explained 81.49% and 10.67% of the total variance in the dataset, respectively. The blue ellipse denotes the positive correlation between the resistance to two antibiotics from a different antibiotic family with the highest Pearson-correlation coefficient.

**Figure 10:**
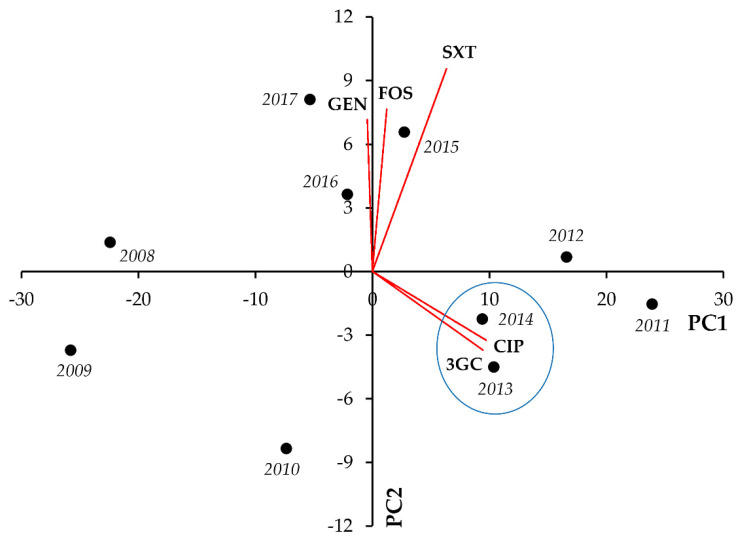
Principal component analysis (PCA) of resistance data for inpatient PPM group UTI isolates among five indicator antibiotics (2008–2017). PC1 and PC2 axes explained 81.85% and 8.35% of the total variance in the dataset, respectively. The blue ellipse denotes the positive correlation between the resistance to two antibiotics from a different antibiotic family with the highest Pearson-correlation coefficient.

**Figure 11:**
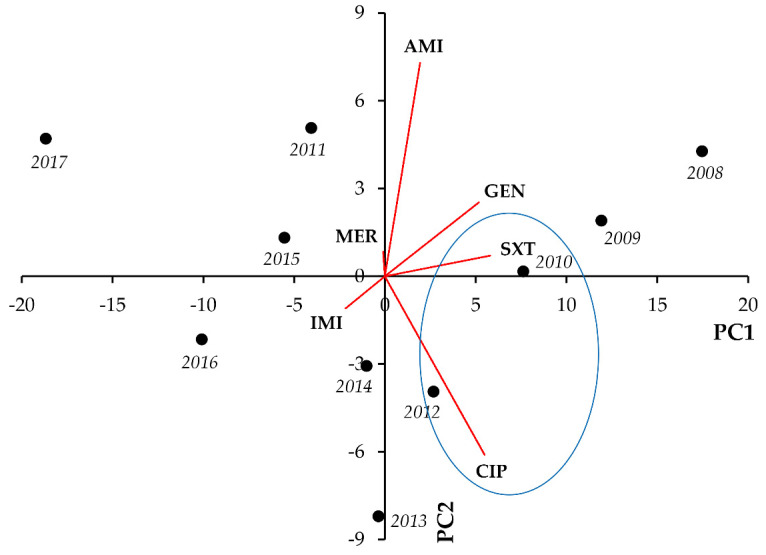
Principal component analysis (PCA) of resistance data for inpatient *Acinetobacter* spp. UTI isolates among six indicator antibiotics (2008–2017). PC1 and PC2 axes explained 75.02% and 12.51% of the total variance in the dataset, respectively. The blue ellipse denotes the positive correlation between the resistance to two antibiotics from a different antibiotic family with the highest Pearson-correlation coefficient.

**Figure 12:**
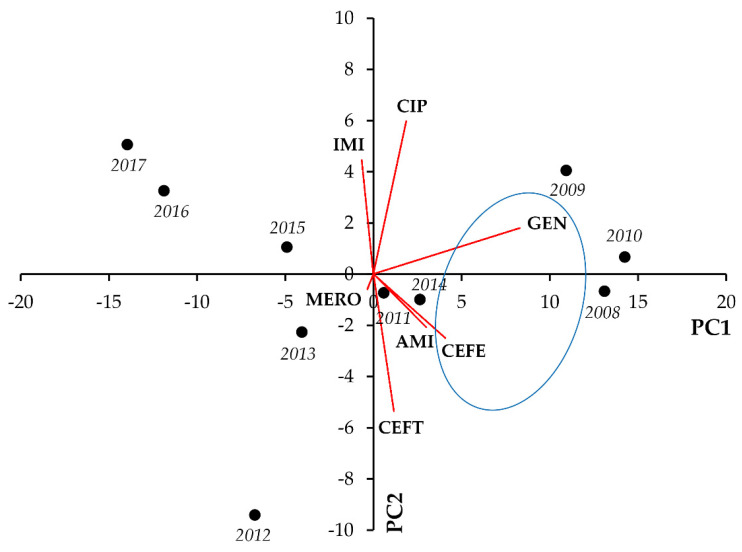
Principal component analysis (PCA) of resistance data for inpatient *Pseudomonas* spp. UTI isolates among seven indicator antibiotics (2008–2017). PC1 and PC2 axes explained 74.41% and 12.08% of the total variance in the dataset, respectively. The blue ellipse denotes the positive correlation between the resistance to two antibiotics from a different antibiotic family with the highest Pearson-correlation coefficient.

The results of the PCA analyses and the corresponding correlation matrices for the data from inpatient UTI isolates are presented in Figure 7, Figure 8, Figure 9, Figure 10, Figure 11 and Figure 12 and Table 7, Table 8, Table 9, Table 10, Table 11 and Table 12, respectively. Similarly to the case of outpatient isolates, strong positive correlation was seen between CIP-GEN (r = 0.9602; *p* < 0.001), CIP-3GC (r = 0.7476; *p* = 0.012), and GEN-3GC (r = 0.8099; *p* = 0.004), whereas strong negative correlation was seen for FOS and NIT resistance (r = −0.7437; *p* = 0.014) (Figure 7, Table 7).

In *Klebsiella* spp., strong positive correlation was seen between resistance to CIP-SXT (r = 0.8144; *p* = 0.004) and CIP-FOS (r = 0.7758; *p* < 0.001) (Figure 8, Table 8).

For members of the *Citrobacter-Enterobacter-Serratia* group, the resistance to one indicator antibiotic showed a strong correlation with resistance to all other indicator antibiotics in almost all cases (strongest correlation was observed for 3GC-CIP [r = 0.8716; *p* = 0.001] and 3GC-SXT [r = 0.8962; *p* < 0.001], respectively). Nonetheless, correlations among FOS and the other antimicrobials were exclusively negative (Figure 9, Table 9).

In case of the *Proteus-Providencia-Morganella* group, strong and significant positive correlations were seen between CIP-3GC (r = 0.8921; *p* < 0.001), CIP-SXT (r = 0.8007; *p* = 0.005), and 3GC-SXT (r = 0.7373; *p* = 0.015) (Figure 10, Table 10).

In *Acinetobacter* spp., most notably, we observed strong and significant negative correlations between IMI-CIP (r = −0.6785; *p* = 0.031), IMI-GEN (r = −0.7102; *p* = 0.021), and IMI-SXT resistance (r = −0.8416; *p* = 0.002), whereas positive correlations were noted for CIP-GEN (r = 0.7096; *p* = 0.022) and CIP-SXT resistance (r = 0.7970; *p* = 0.006), respectively (Figure 11, Table 11).

Lastly, in Pseudomonas spp., notable positive correlations were identified for GEN-CEFE (r = 0.8024; *p* = 0.004 and GEN-AMI (r = 0.8565; *p* = 0.002), whereas negative correlation was seen between CEFT and IMI resistance (r = −0.6612; *p* = 0.037) (Figure 12, Table 12).

### 3.4. Discussion

Antimicrobial resistance (AMR) is one of the most worrisome threats humanity has to face in the 21st century, which has been recognized by many government leaders and trans-national organizations [42]. Due to the extensive resistance in many of the Gram-negative bacteria, these pathogens are considered as a priority for R&D and antimicrobial drug discovery platforms, to facilitate the development of new antibiotics [43]. In addition, continuous monitoring of resistance rates in a given institution or geographical region is another necessity to successfully address AMR globally [44]. Bacteria may present with the MDR/XDR phenotype through the contribution of a variety of mechanisms, including both intrinsic and acquired resistance [45]. The detailed knowledge of intrinsic resistance mechanisms in Gram-negative bacteria is critical for clinicians and infectious disease specialists, as they need to be considered even in cases of otherwise susceptible isolates. A summary of intrinsic resistance mechanisms seen in relevant Gram-negative bacteria is presented in Table 13.

Molecular tests and sequencing-based platforms are now considered as the gold-standard for AMR surveillance, providing detailed information on what kind of resistance-determinants may be found in a given bacterial isolate, as well as the type of genetic information (chromosomal or MGE), which influences the possibility of rapid dissemination, especially in a nosocomial environment [30,47]. However, these technologies are not yet available to most routine clinical laboratories with high turnovers of clinical material. Moreover, the identification of a resistance gene alone does not predict the relationship between genotype and phenotype (i.e., the expression level of the gene), often leading to discrepancies [48]. For this reason, the standardized collection of phenotypic resistance data, and the introduction of Big Data analytic methods into AMR surveillance may be a viable alternative for molecular surveillance (and to maintain “regional” antibiograms), especially in low-resource settings [49]. Multivariate analyses of large datasets involving bacteria (e.g., resistance rates, expression of virulence factors, biofilm-formation), such as PCA and correlation matrices, have been performed [39]. For example, Amsalu et al. analyzed phenotypic data and sequencing in *n* = 147 *P. aeruginosa* to assess the correlation between the resistance to biocide-resistance, and their results suggested that biocide resistance showed a significant positive correlation between biocide resistance and phenotypic resistance to fluoroquinolones, cephalosporins, and aminoglycosides [31]. Zhang et al. identified significant positive correlation among resistance and the presence of virulence genes associated with the extra-intestinal pathogenic (ExPEC) pathotype of *E. coli* isolated from healthy ducks [50]. Zhang et al. analyzed phenotypic and genotypic susceptibility data of foodborne pathogens from the NCBI Pathogen Detection Isolates Browser (NPDIB) database, corresponding to six US states, using PCA and hierarchical clusters [40]. They found that isolates from states in geographic proximity (Pennsylvania, New York, and Maryland) shared more similar resistance genes, and overall, the following ten genes were the most common contributors to the MDR phenotype: *aadA*; *aph(3”)*; *aph(3”)-Ib*; *aph(6)-I*; *aph(6)-Id*; *bla*; *blaCMY*; *tet*; *tet(A)*; and *sul2* [40]. Li et al. utilized the same NPDIB database to assess phenotypic and genotypic susceptibility data from six different countries (Australia, Brazil, China, South Africa, the UK, and the US), and they have shown that geographical proximity was an important factor in identifying common resistance genes, and some resistance-determinants (i.e., *aph(3”)-Ib*, *aph(6)-Id*, *blaTEM-1*, and *qacEδ1*) were shared among all six countries. The authors have proposed, based on these historical resistance data, potential avenues for the spreading of antimicrobial resistance genes [51]. Mandal et al. studied the correlation between the multiple antibiotic resistance (MAR) phenotype and heavy metal (Hg^2+^, Cd^2+^, Cr^2+^, and Cu^2+^) resistance in *E. coli* and non-fermenting Gram-negative bacteria isolated from sewage wastewater, and found significant correlation between MAR indices and heavy metal tolerance [52].

UTIs—especially in primary care settings—are treated empirically in most cases, due to the predictable range of pathogens implicated in these infections. Traditionally, NIT, FOS, NIT, and pivmecillinam are recommended for uncomplicated UTIs, whereas for more severe cases, fluoroquinolones and II-III. generation cephalosporins are often prescribed [53]. However, with the increase in MDR rates, these abovementioned drugs have decreased effectiveness, and the inappropriate empiric therapy of these infections leads to selection pressure, an additional burden for the patient and the healthcare system [25,54]. Though from an antimicrobial stewardship point of view, the management of UTIs may be considered as “low hanging fruit”, with the mortality rate of these infections being much lower compared to invasive infections (e.g., sepsis, pneumonia) caused by the same bacteria, due to their high incidence and large patient population affected, these infections still have plenty of room for significant interventions to be made in the prudent and thoughtful utilization of antibiotics [55]. As a part of our secondary study, PCA and correlation analyses were performed, corresponding to resistance data of >30,000 UTIs, representing taxonomically-diverse Gram-negative pathogens, spanning over a 10-year long surveillance period from our tertiary-care hospital in Hungary. Our aim was to establish the co-occurrence of phenotypic resistance to indicator antibiotics in these isolates, to potentially uncover associations that were previously unaddressed, and to identify which resistances are the most relevant contributors to the development of the MDR phenotype (i.e., resistance to one agent in at least three different antibiotic groups [56]) in our setting. This information may direct therapeutic decisions to avoid the extensive use of some antimicrobials, decreasing unwanted selection pressure by these drugs.

This present study is a continuation of a comprehensive characterization of 10-years’ worth of UTI resistance data in Southern Hungary [33]. Our data analyses have revealed some strong associations and co-occurrences of phenotypic resistance: in outpatient *E. coli* isolates, CIP resistance was a principal factor, commonly associated with GEN and SXT resistance, whereas in inpatient isolates, GEN resistance was the most common denominator, strongly associated with CIP, SXT, and resistance of 3GCs. In inpatient and outpatient *E. coli* alike, a strong negative correlation was seen between FOS and NIT resistance, both being commonly-used antimicrobials in the treatment of uncomplicated UTIs [54]. Interestingly, a slightly different picture was observed in outpatient and inpatient *Klebsiella* spp.: though in both cases, cohesion was seen in CIP resistance, in outpatient isolates, strong positive correlations were seen between CIP-GEN and CIP-3GC resistance, whereas for inpatient isolates, associations of CIP-SXT and CIP-FOS co-resistance were the most relevant. In the CES group isolates, strong correlations were seen among the resistance to most antimicrobials included: in the outpatient group, GEN resistance was the strongest common denominator, whereas in the inpatient group, 3GC resistance was the most relevant. Interestingly, in inpatient isolates, strong negative correlation was shown between FOS resistance and resistance to all other indicator antibiotics. In the PPM group isolates, CIP was the common axis of co-resistance, showing very strong, positive correlation with SXT in both groups (in addition to FOS and 3GC in the outpatient and inpatient group, respectively). In outpatient *Acinetobacter* spp. isolates, CIP showed strong and positive correlations with all other indicator antibiotics, in addition to IMI showing common co-occurrence with the two aminoglycoside drugs (GEN and AMI). In inpatient isolates, the significant co-occurrence of CIP with other antibiotics was less common, whereas SXT resistance showed strong positive associations with the members of a variety of antibiotic groups (i.e., IMI, GEN and CIP). In outpatient *Pseudomonas* spp., an interesting distinction was observed: strong positive correlation was seen between CEFE and other antibiotics (IMI, MER and GEN), whereas strong negative correlation was detected with CEFT and the same antimicrobials. In inpatient isolates, CEFE resistance showed strong positive co-occurrence with the two aminoglycoside drugs, whereas strong negative correlation was observed between CEFT-IMI. Co-occurrence of CIP resistance was not as relevant in *Pseudomonas* spp. as in other Gram-negative bacteria. Unsurprisingly, both in *Pseudomonas* spp. and *Acinetobacter* spp., strong and positive correlation was shown between the resistance to the two aminoglycoside drugs (GEN and AMI). The data presented herein may prove to be useful in complementing already existing antimicrobial stewardship interventions and hospital antibiograms. Based on our results, we have shown that—from our data—no overarching conclusions may be drawn for Gram-negative bacteria as a whole. As the presence of resistance to each individual indicator antibiotic had varying relevance in different taxonomic groups, this needs to be addressed accordingly. In addition, variations have occurred even between inpatient and outpatient isolates of the same taxonomic unit, which is most probably due to the different set of relevant antimicrobials prescribed in outpatient clinics and inpatient departments (with the opportunity to use antimicrobials in intravenous infusion form in the latter setting), as not all antibiotics are equally relevant in all patient groups (based on age or underlying conditions) [57].

Age and gender of the patients affected by UTIs has been suggested as an important epidemiological factor in forecasting resistance rates in urinary pathogens, i.e., with the increasing age of patients, resistance rates may also show an increasing trend [58]. Although we did not ascertain this correlation as a part of this study, our previous studies corresponding to the UTIs described in patients aged ≥65 years of age, and in male patients (whom are affected by complicated UTIs, usually in advanced ages) have both shown higher rates of resistance to UTI-specific drugs (nitrofurantoin, fosfomycin), 3GCs, fluoroquinolones, and a higher prevalence of MDR isolates overall, compared to the general population [59,60]. The burden of UTIs in healthcare-associated infections has been highlighted by a nosocomial surveillance study in a ~1600-bed hospital in Eastern Hungary, where 21.1% of such infections were UTIs during the study period (2004–2006) [61]. In addition, a study by Bánhidy et al. reported that 5.7% of mothers were affected by UTIs during pregnancy, and these pregnancies had a higher proportion of preterm births (10.4% vs. 9.1%) [62]. The study of Szász et al. included the analysis of uropathogens from seven inpatient clinics of the Semmelweis University (Budapest) between the years 2006 and 2008, and the species distribution reported was similar to our study (Gram-negative bacteria in the majority, *E. coli*: 34–54%, *Klebsiella* spp.: 3–11%, *Pseudomonas* spp.: 3–9%, the PPM group: 3.5–8%, and *Enterobacter* spp.: 2–6%), however, resistance rates reported were considerably lower in their paper [63]. The study of Illesy et al. described the prevalence of infections in kidney transplant recipients between 2010 and 2015 at the Faculty of Medicine, University of Debrecen: 69.7% of patients developed an infection, 79.3% (*n* = 88) of the infections were UTIs (caused by *E. coli*, *Enterococcus faecalis* and *Klebsiella* spp.), and 19.8% of the infections were MDR [64]. Finally, the recently-published microbial surveillance study of Magyar et al. described the UTI epidemiology at the urology department of the Jahn Ferenc South Pest Teaching Hospital (Budapest) between 2004 and 2015. *E. coli* and *E. faecalis* were shown to be the most common uropathogens, however, a slow but noticeable growth in the rates of *P. mirabilis* and *P. aeruginosa* was also seen throughout the study. Similar to our results, this study also highlighted the retained effectiveness of carbapenems and polymyxin B for Enterobacterales, and high levels of fluoroquinolone resistance in most bacteria. In contrast to our study, they have shown very low resistance rates to fosfomycin (0–15%) and nitrofurantoin (<2%) in *E. coli*, and high resistance rates to 3GCs in Klebsiella spp. (owing to the high prevalence of ESBL-producers) [65].

It must be addressed that the rates of developing resistance (either through spontaneous mutations or through acquiring MGEs via horizontal gene transfer) may be slightly different between distinct antibiotic families [66]: for example, there are chromosomally-encoded β-lactamases in Enterobacterales and ESBLs, and now carbapenemases are located on MGEs (plasmids, integrons, or transposons), and are epidemiologically much more relevant, due to the risk of their rapid dissemination. For aminoglycoside-inactivating enzymes, chromosomal carriage and MGEs are equally important, whereas for high-level phenotypic fluoroquinolone-resistance to occur, often the presence of more than resistance determinants (chromosomal and plasmid-mediated resistance, affecting DNA gyrase and topoisomerase IV, coupled with alterations in outer membrane proteins or overexpression of efflux pumps) is needed [67,68]. On the other hand, colistin-resistance and plasmid-borne colistin resistance (encoded by the mcr genes) were first described in 2015 in *E. coli* and *K. pneumoniae* [69]. It has been extensively described—both in Hungary and in other countries—that acquiring plasmid-borne fluoroquinolone resistance is a critical step towards the development of MDR in both Enterobacterales positive or negative for ESBL-production (as also demonstrated by our data analysis) [70]. Subsequently, these isolates will present with chromosomal or plasmid-mediated aminoglycoside-resistance and resistance to other ancillary antibiotics (such as nitrofurantoin, fosfomycin, trimethoprim-sulfamethoxazole, and next-generation tetracycline-derviatives) [71]. In the end, the process ends with infections that may only be treated by carbapenems, novel β-lactam/β-lactamase-inhibitors, and colistin [72]. Tracing the steps towards the development of the MDR phenotype in non-fermenters (*Acinetobacter* spp., *Pseudomonas* spp.) is not as straightforward. Though resistance against fluoroquinolones and aminoglycosides may undoubtedly occur in these pathogens through similar mechanisms seen in Enterobacterales, the mechanisms and contribution of resistance to β-lactam antibiotics in non-fermenters is more diverse, with downregulation or absence of the OprD porins, efflux pump-overexpression, and penicillin-binding protein (PBP) modifications also having pronounced roles, in addition to β-lactamases [73,74,75]. Moreover, the presence of genotypic resistance mechanisms in these bacteria may affect the in vitro susceptibility of individual antibiotics—even in the same family of antibiotics—differently (e.g., often resulting in isolates non-susceptible to meropenem, but not imipenem) [76,77]. Our study highlights the importance of accumulating large datasets (either phenotypic or genotypic in nature) originating from microbiological isolates, and taking advantage of them in secondary data analyses for the purposes of basic science or clinical practice [78].

## 4. Conclusions

As the threat of AMR is reaching critical levels worldwide, the importance of timely surveillance regarding resistance rates—both for epidemiological purposes and to aid the selection of empiric therapy for clinicians—must be one of the key priorities of relevant stakeholders. This data collection may range from the simple collection of phenotypic resistance rates (to create a hospital antibiogram) to the extensive utilization of sequencing methods to monitor dominant MDR clones and genotypic resistance-determinants in the region, depending on the availability of the methods and funding. Though many microbiology laboratories (often serving large geographical regions) simply do not have the resources to maintain advanced sequencing platforms, they may possess considerably-sized longitudinal resistance data, which could provide valuable insights for AMR surveillance on a local and a national level. Our paper—detailing the co-occurrence and correlation of phenotypic resistance in Gram-negative UTIs for 10 years—may be considered a case study for the feasibility of standardized phenotypic resistance data collection and the use of data mining (considering resistance data as “Big Data”) to be a cost-friendly alternative for molecular surveillance in resource-scarce settings. The syndrome-specific analyses of large resistance datasets (spanning over long periods of time) may show the decreasing relevance of some antibiotic groups in the treatment of the given bacterial infection, in addition to showing the tendency of resistances to major antibiotic groups presenting in parallel, acting as a “compass” to avoid therapeutic failure at the bedside.

## Figures and Tables

**Table 1:** Correlation matrix of resistance data for outpatient *E. coli* UTI isolates among six indicator antibiotics (2008–2017).

	**CIP**	**NIT**	**GEN**	**SXT**	**3GC**	**FOS**	
**CIP**	X	0.2826	*0.049*	*0.002*	0.3693	0.3489	Statistical significance (*p*=)
**NIT**	−0.3772	X	0.7205	0.1918	0.1893	*0.019*	
**GEN**	0.6342	0.1299	X	0.051	0.6080	0.7434	
**SXT**	0.8474	−0.4501	0.6289	X	0.078	0.4530	
**3GC**	0.3188	−0.4524	0.1855	0.5798	X	0.9514	
**FOS**	0.3318	−0.7183	0.1189	0.2686	−0.022	X	
	Pearson-correlation coefficient (r=)						X

Green cells denote Pearson-correlation coefficients representing strong (0.5 < |r| < 0.85) and statistically significant (*p* < 0.05) correlations; blue cells denote Pearson-correlation coefficients representing strong (0.5 < |r| < 0.85), but statistically non-significant (*p* ≥ 0.05) correlations; white cells denote Pearson-correlation coefficients representing moderate or weak (|r| < 0.5), statistically non-significant (*p* ≥ 0.05) correlations. Legend: CIP: ciprofloxacin; NIT: nitrofurantoin; GEN: gentamicin; SXT: trimethoprim-sulfamethoxazole; 3GC: third-generation cephalosporin; FOS: fosfomycin.

**Table 2:** Correlation matrix of resistance data for outpatient *Klebsiella* spp. UTI isolates among five indicator antibiotics (2008–2017).

	**CIP**	**GEN**	**SXT**	**3GC**	**FOS**	
**CIP**	X	*0.014*	0.9653	*0.006*	0.8964	Statistical significance (*p*=)
**GEN**	0.7428	X	0.8188	0.1723	0.4207	
**SXT**	0.0158	0.0834	X	0.4319	0.7445	
**3GC**	0.7976	0.4679	0.2808	X	0.4336	
**FOS**	0.0475	−0.2874	0.1184	0.2798	X	
	Pearson-correlation coefficient (r=)					X

Green cells denote Pearson-correlation coefficients representing strong (0.5 < |r| < 0.85) and statistically significant (*p* < 0.05) correlations; white cells denote Pearson-correlation coefficients representing moderate or weak (|r| < 0.5), statistically non-significant (*p* ≥ 0.05) correlations. Legend: CIP: ciprofloxacin; GEN: gentamicin; SXT: trimethoprim-sulfamethoxazole; 3GC: third-generation cephalosporin; FOS: fosfomycin.

**Table 3:** Correlation matrix of resistance data for outpatient CES group UTI isolates among five indicator antibiotics (2008–2017).

	**CIP**	**3GC**	**GEN**	**SXT**	**FOS**	
**CIP**	X	*0.035*	*<0.001*	*0.017*	0.3837	Statistical significance (*p*=)
**3GC**	0.6683	X	*0.004*	0.068	0.1181	
**GEN**	0.9326	0.8144	X	*0.004*	0.1524	
**SXT**	0.7266	0.5974	0.8152	X	*0.027*	
**FOS**	0.3098	0.5263	0.4881	0.6895	X	
	Pearson-correlation coefficient (r=)					X

Orange cells denote Pearson-correlation coefficients representing very strong (|r| ≥ 0.85) and statistically significant (*p* < 0.05) correlations; green cells denote Pearson-correlation coefficients representing strong (0.5 < |r| < 0.85) and statistically significant (*p* < 0.05) correlations; blue cells denote Pearson-correlation coefficients representing strong (0.5 < |r| < 0.85), but statistically non-significant (*p* ≥ 0.05) correlations; white cells denote Pearson-correlation coefficients representing moderate or weak (|r| < 0.5), statistically non-significant (*p* ≥ 0.05) correlations. Legend: CIP: ciprofloxacin; GEN: gentamicin; SXT: trimethoprim-sulfamethoxazole; 3GC: third-generation cephalosporin; FOS: fosfomycin.

**Table 4:** Correlation matrix of resistance data for outpatient PPM group UTI isolates among five indicator antibiotics (2008–2017).

	**CIP**	**3GC**	**GEN**	**SXT**	**FOS**	
**CIP**	X	0.051	0.4931	*<0.001*	*<0.001*	Statistical significance (*p*=)
**3GC**	0.6296	X	0.5959	0.1318	0.051	
**GEN**	0.2461	−0.1916	X	0.4162	0.4188	
**SXT**	0.9251	0.5103	0.2901	X	*<0.001*	
**FOS**	0.8656	0.6304	0.2886	0.8992	X	
	Pearson-correlation coefficient (r=)					X

Orange cells denote Pearson-correlation coefficients representing very strong (|r| ≥ 0.85) and statistically significant (*p* < 0.05) correlations; blue cells denote Pearson-correlation coefficients representing strong (0.5 < |r| < 0.85), but statistically non-significant (*p* ≥ 0.05) correlations; white cells denote Pearson-correlation coefficients representing moderate or weak (|r| < 0.5), statistically non-significant (*p* ≥ 0.05) correlations. Legend: CIP: ciprofloxacin; GEN: gentamicin; SXT: trimethoprim-sulfamethoxazole; 3GC: third-generation cephalosporin; FOS: fosfomycin.

**Table 5:** Correlation matrix of resistance data for outpatient *Acinetobacter* spp. UTI isolates among six indicator antibiotics (2008–2017).

	**CIP**	**IMI**	**MER**	**GEN**	**AMI**	**SXT**	
**CIP**	X	*0.041*	*0.039*	*0.026*	*0.019*	0.1009	Statistical significance (*p*=)
**IMI**	0.6507	X	0.2298	*0.001*	*0.002*	0.054	
**MER**	0.6554	0.4176	X	0.4330	0.3727	0.7539	
**GEN**	0.6945	0.8620	0.2802	X	*<0.001*	*0.048*	
**AMI**	0.7159	0.8375	0.3166	0.9838	X	*0.035*	
**SXT**	0.5482	0.6233	0.1139	0.6361	0.6669	X	
	Pearson-correlation coefficient (r=)						X

Orange cells denote Pearson-correlation coefficients representing very strong (|r| ≥ 0.85) and statistically significant (*p* < 0.05) correlations; green cells denote Pearson-correlation coefficients representing strong (0.5 < |r| < 0.85) and statistically significant (*p* < 0.05) correlations; blue cells denote Pearson-correlation coefficients representing strong (0.5 < |r| < 0.85), but statistically non-significant (*p* ≥ 0.05) correlations; white cells denote Pearson-correlation coefficients representing moderate or weak (|r| < 0.5), statistically non-significant (*p* ≥ 0.05) correlations. Legend: CIP: ciprofloxacin; IMI: imipenem; MER: meropenem; GEN: gentamicin; AMI: amikacin; SXT: trimethoprim-sulfamethoxazole.

**Table 6:** Correlation matrix of resistance data for outpatient *Pseudomonas* spp. UTI isolates among seven indicator antibiotics (2008–2017).

	**CIP**	**IMI**	**MER**	**CEFT**	**CEFE**	**GEN**	**AMI**	
**CIP**	X	0.5507	0.3572	0.1489	0.1359	0.3528	0.1145	Statistical significance (*p*=)
**IMI**	−0.2151	X	0.0991	*0.007*	*0.001*	*0.038*	0.3386	
**MER**	0.3265	−0.5505	X	0.1316	*0.016*	0.3288	0.5279	
**CEFT**	−0.4917	−0.7879	−0.5105	X	*0.002*	0.063	0.1911	
**CEFE**	0.5057	0.8617	0.7338	−0.8376	X	*0.028*	0.1139	
**GEN**	0.3293	−0.6595	0.3451	−0.6056	0.6857	X	*<0.001*	
**AMI**	0.5307	−0.3386	0.2272	−0.4507	0.5315	0.8745	X	
	Pearson-correlation coefficient (r=)							X

Orange cells denote Pearson-correlation coefficients representing very strong (|r| ≥ 0.85) and statistically significant (*p* < 0.05) correlations; green cells denote Pearson-correlation coefficients representing strong (0.5 < |r| < 0.85) and statistically significant (*p* < 0.05) correlations; blue cells denote Pearson-correlation coefficients representing strong (0.5 < |r| < 0.85), but statistically non-significant (*p* ≥ 0.05) correlations; white cells denote Pearson-correlation coefficients representing moderate or weak (|r| < 0.5), statistically non-significant (*p* ≥ 0.05) correlations. Legend: CIP: ciprofloxacin; IMI: imipenem; MER: meropenem; CEFT: ceftazidime; CEFE: cefepime; GEN: gentamicin; AMI: amikacin.

**Table 7:** Correlation matrix of resistance data for inpatient *E. coli* UTI isolates among six indicator antibiotics (2008–2017).

	**CIP**	**NIT**	**GEN**	**SXT**	**3GC**	**FOS**	
**CIP**	X	0.1057	*<0.001*	0.1865	*0.012*	0.2239	Statistical significance (*p*=)
**NIT**	−0.5418	X	0.1449	0.0942	0.1694	*0.014*	
**GEN**	0.9602	−0.4959	X	0.1503	*0.004*	0.3587	
**SXT**	0.4549	−0.5572	0.4902	X	0.068	0.2854	
**3GC**	0.7476	−0.4711	0.8099	0.5970	X	0.6951	
**FOS**	0.4224	−0.7437	0.3255	0.3752	0.1422	X	
	Pearson-correlation coefficient (r=)						X

Orange cells denote Pearson-correlation coefficients representing very strong (|r| ≥ 0.85) and statistically significant (*p* < 0.05) correlations; green cells denote Pearson-correlation coefficients representing strong (0.5 < |r| < 0.85) and statistically significant (*p* < 0.05) correlations; blue cells denote Pearson-correlation coefficients representing strong (0.5 < |r| < 0.85), but statistically non-significant (*p* ≥ 0.05) correlations; white cells denote Pearson-correlation coefficients representing moderate or weak (|r| < 0.5), statistically non-significant (*p* ≥ 0.05) correlations. Legend: CIP: ciprofloxacin; NIT: nitrofurantoin; GEN: gentamicin; SXT: trimethoprim-sulfamethoxazole; 3GC: third-generation cephalosporin; FOS: fosfomycin.

**Table 8:** Correlation matrix of resistance data for inpatient *Klebsiella* spp. UTI isolates among five indicator antibiotics (2008–2017).

	**CIP**	**GEN**	**SXT**	**3GC**	**FOS**	
**CIP**	X	0.2592	*0.004*	0.060	*<0.001*	Statistical significance (*p*=)
**GEN**	−0.3946	X	0.9287	0.5970	0.092	
**SXT**	0.8144	−0.0326	X	0.054	0.1501	
**3GC**	0.6119	0.1910	0.6240	X	0.3655	
**FOS**	0.7758	−0.5599	0.4904	0.3212	X	
	Pearson-correlation coefficient (r=)					X

Green cells denote Pearson-correlation coefficients representing strong (0.5 < |r| < 0.85) and statistically significant (*p* < 0.05) correlations; white cells denote Pearson-correlation coefficients representing moderate or weak (|r| < 0.5), statistically non-significant (*p* ≥ 0.05) correlations. Legend: CIP: ciprofloxacin; GEN: gentamicin; SXT: trimethoprim-sulfamethoxazole; 3GC: third-generation cephalosporin; FOS: fosfomycin.

**Table 9:** Correlation matrix of resistance data for inpatient CES group UTI isolates among five indicator antibiotics (2008–2017).

	**CIP**	**3GC**	**GEN**	**SXT**	**FOS**	
**CIP**	X	*0.001*	*0.012*	*0.049*	*0.035*	Statistical significance (*p*=)
**3GC**	0.8716	X	*0.008*	*<0.001*	*0.043*	
**GEN**	0.7509	0.7738	X	0.067	*0.039*	
**SXT**	0.6345	0.8962	0.5989	X	0.2919	
**FOS**	−0.6664	−0.6469	−0.6566	−0.3705	X	
	Pearson-correlation coefficient (r=)					X

Orange cells denote Pearson-correlation coefficients representing very strong (|r| ≥ 0.85) and statistically significant (*p* < 0.05) correlations; green cells denote Pearson-correlation coefficients representing strong (0.5 < |r| < 0.85) and statistically significant (*p* < 0.05) correlations; blue cells denote Pearson-correlation coefficients representing strong (0.5 < |r| < 0.85), but statistically non-significant (*p* ≥ 0.05) correlations; white cells denote Pearson-correlation coefficients representing moderate or weak (|r| < 0.5), statistically non-significant (*p* ≥ 0.05) correlations. Legend: CIP: ciprofloxacin; GEN: gentamicin; SXT: trimethoprim-sulfamethoxazole; 3GC: third-generation cephalosporin; FOS: fosfomycin.

**Table 10:** Correlation matrix of resistance data for inpatient PPM group UTI isolates among five indicator antibiotics (2008–2017).

	**CIP**	**3GC**	**GEN**	**SXT**	**FOS**	
**CIP**	X	*<0.001*	0.6076	*0.005*	0.4827	Statistical significance (*p*=)
**3GC**	0.8921	X	0.8589	*0.015*	0.3256	
**GEN**	−0.1857	−0.0648	X	0.9475	0.2545	
**SXT**	0.8007	0.7373	−0.024	X	0.068	
**FOS**	0.2519	0.3472	0.3982	0.5966	X	
	Pearson-correlation coefficient (r=)					X

Orange cells denote Pearson-correlation coefficients representing very strong (|r| ≥ 0.85) and statistically significant (*p* < 0.05) correlations; blue cells denote Pearson-correlation coefficients representing strong (0.5 < |r| < 0.85), but statistically non-significant (*p* ≥ 0.05) correlations; white cells denote Pearson-correlation coefficients representing moderate or weak (|r| < 0.5), statistically non-significant (*p* ≥ 0.05) correlations. Legend: CIP: ciprofloxacin; GEN: gentamicin; SXT: trimethoprim-sulfamethoxazole; 3GC: third-generation cephalosporin; FOS: fosfomycin.

**Table 11:** Correlation matrix of resistance data for inpatient *Acinetobacter* spp. UTI isolates among six indicator antibiotics (2008–2017).

	**CIP**	**IMI**	**MER**	**GEN**	**AMI**	**SXT**	
**CIP**	X	*0.031*	0.4486	*0.022*	0.5698	*0.006*	Statistical significance (*p*=)
**IMI**	−0.6785	X	0.3804	*0.021*	0.083	*0.002*	
**MER**	−0.2711	0.3119	X	0.9967	0.8073	0.8697	
**GEN**	0.7096	−0.7102	−0.001	X	0.1456	*0.007*	
**AMI**	0.2051	−0.5735	−0.0888	0.4952	X	0.1561	
**SXT**	0.7970	−0.8416	0.0598	0.7857	0.4843	X	
	Pearson-correlation coefficient (r=)						X

Green cells denote Pearson-correlation coefficients representing strong (0.5 < |r| < 0.85) and statistically significant (*p* < 0.05) correlations; blue cells denote Pearson-correlation coefficients representing strong (0.5 < |r| < 0.85), but statistically non-significant (*p* ≥ 0.05) correlations; white cells denote Pearson-correlation coefficients representing moderate or weak (|r| < 0.5), statistically non-significant (*p* ≥ 0.05) correlations. Legend: CIP: ciprofloxacin; IMI: imipenem; MER: meropenem; GEN: gentamicin; AMI: amikacin; SXT: trimethoprim-sulfamethoxazole.

**Table 12:** Correlation matrix of resistance data for inpatient *Pseudomonas* spp. UTI isolates among seven indicator antibiotics (2008–2017).

	**CIP**	**IMI**	**MER**	**CEFT**	**CEFE**	**GEN**	**AMI**	
**CIP**	X	0.7249	0.6326	0.7215	0.4219	0.091	0.5413	Statistical significance (*p*=)
**IMI**	0.1278	X	0.9402	*0.037*	0.7006	0.5551	0.1633	
**MER**	−0.1731	0.027	X	0.9351	0.8871	0.5665	0.3523	
**CEFT**	−0.1295	−0.6612	0.0297	X	0.072	0.3534	0.1695	
**CEFE**	0.2867	−0.1396	0.0517	0.5910	X	*0.004*	*0.048*	
**GEN**	0.5614	−0.2128	−0.2068	0.3289	0.8204	X	*0.002*	
**AMI**	0.2201	−0.4771	−0.3296	0.4709	0.6372	0.8565	X	
	Pearson-correlation coefficient (r=)							X

Orange cells denote Pearson-correlation coefficients representing very strong (|r| ≥ 0.85) and statistically significant (*p* < 0.05) correlations; green cells denote Pearson-correlation coefficients representing strong (0.5 < |r| < 0.85) and statistically significant (*p* < 0.05) correlations; blue cells denote Pearson-correlation coefficients representing strong (0.5 < |r| < 0.85), but statistically non-significant (*p* ≥ 0.05) correlations; white cells denote Pearson-correlation coefficients representing moderate or weak (|r| < 0.5), statistically non-significant (*p* ≥ 0.05) correlations. Legend: CIP: ciprofloxacin; IMI: imipenem; MER: meropenem; CEFT: ceftazidime; CEFE: cefepime; GEN: gentamicin; AMI: amikacin.

**Table 13:** Summary of intrinsic resistance exhibited by Gram-negative bacteria included in this study (adapted from [25,45,46]).

All Gram-negative bacteria	glycopeptides (e.g., vancomycin), lincosamides (e.g., clindamycin), oxazolidinones (linezolid, tedizolid), streptogramins (quinpristin-dalfopristin), macrolides (e.g., azithromycin), daptomycin, tetracycline
*Escherichia coli*	-
*Klebsiella* spp.	ampicillin
*Citrobacter-Enterobacter-Serratia* group	CES: aminopenicillins, aminopenicillin/β-lactamase-inhibitor combinations, I–II. generation cephalosporins; *Serratia* spp.: nitrofurantoin, doxycycline, polymyxin B, colistin, aminoglycosides (except: streptomycin, amikacin)
*Proteus-Providencia-Morganella* group	resistance: aminopenicillins, aminopenicillin/β-lactamase-inhibitor combinations, I–II. generation cephalosporins (except for: *P. mirabilis*), nitrofurantoin, doxycycline, polymyxin B, colistin; reduced susceptibility: imipenem
*Acinetobacter* spp.	aminopenicillins, aminopenicillin/β-lactamase-inhibitor combinations, I–III. generation cephalosporins, ertapenem, aztreonam, nitrofurantoin, doxycycline, fosfomycin
*Pseudomonas* spp.	aminopenicillins, aminopenicillin/β-lactamase-inhibitor combinations, I–II. generation cephalosporins, orally administered III. generation cephalosporins, ertapenem, trimethoprim-sulfamethoxazole, rifampin, nitrofurantoin, doxycycline, tigecycline

## Data Availability

The primary data used during the analyses of this study may be found in the publication of Gajdács et al. (https://www.mdpi.com/2075-1729/10/2/16, accessed on 8 August 2021).
